# Cancer Pain Experience Through the Lens of Patients and Caregivers: Mixed Methods Social Media Study

**DOI:** 10.2196/41594

**Published:** 2023-07-03

**Authors:** Chiara Filipponi, Mariam Chichua, Marianna Masiero, Davide Mazzoni, Gabriella Pravettoni

**Affiliations:** 1 Applied Research Division for Cognitive and Psychological Science IEO European Institute of Oncology IRCCS Milan Italy; 2 Department of Oncology and Hemato-Oncology University of Milan Milan Italy

**Keywords:** pain, cancer, quality of life, social support, emotion, personality, decision-making

## Abstract

**Background:**

Cancer pain represents a challenge for cancer patients and their family members. Despite progression in pain management, pain is still underreported and undertreated, and there is limited information on the related needs that patients and caregivers may have. Online platforms represent a fundamental tool for research to reveal the unmet needs of these users and their emotions outside the medical setting.

**Objective:**

This study aimed to (1) reveal the unmet needs of both patients and caregivers and (2) detect the emotional activation associated with cancer pain by analyzing the textual patterns of both users.

**Methods:**

A descriptive and quantitative analysis of qualitative data was performed in RStudio v.2022.02.3 (RStudio Team). We analyzed 679 posts (161 from caregivers and 518 from patients) published over 10 years on the “cancer” subreddit of Reddit to identify unmet needs and emotions related to cancer pain. Hierarchical clustering, and emotion and sentiment analysis were conducted.

**Results:**

The language used for describing experiences related to cancer pain and expressed needs differed between patients and caregivers. For patients (agglomerative coefficient=0.72), the large cluster labeled *unmet needs* included the following clusters: (1A) *reported experiences*, with the subclusters (a) *relationship with doctors/spouse* and (b) *reflections on physical features*; and (1B) *changes observed over time*, with the subclusters (a) *regret* and (b) *progress.* For caregivers (agglomerative coefficient=0.80), the main clusters were as follows: (1A) *social support* and (1B) *reported experiences*, with the subclusters (a) *psychosocial challenges* and (b) *grief*. Moreover, comparison between the 2 groups (entanglement coefficient=0.28) showed that they shared a common cluster labeled *uncertainty*. Regarding emotion and sentiment analysis, patients expressed a significantly higher negative sentiment than caregivers (z=−2.14; *P*<.001). On the contrary, caregivers expressed a higher positive sentiment compared with patients (z=−2.26; *P*<.001), with trust (z=−4.12; *P*<.001) and joy (z=−2.03; *P*<.001) being the most prevalent positive emotions.

**Conclusions:**

Our study emphasized different perceptions of cancer pain in patients and caregivers. We revealed different needs and emotional activations in the 2 groups. Moreover, our study findings highlight the importance of considering caregivers in medical care. Overall, this study increases knowledge about the unmet needs and emotions of patients and caregivers, which may have important clinical implications in pain management.

## Introduction

### Background

Cancer pain represents a challenge for cancer patients at different time points of their medical path, from diagnosis to long-term survivorship and palliative care. In a recent meta-analysis [1], cancer pain was demonstrated to be most prevalent after and during anticancer treatments (prevalence rate from 39% to 55%), followed by advanced, metastatic, or terminal disease (66%). According to Dame Cicely Saunders [2,3], pain is not just a physical experience in oncology. It involves all components of human functioning, including psychological, social, and spiritual aspects, resulting in a “total pain” experience.

From a psychological point of view, cancer pain may represent a source of emotional distress, anxiety, depression, fear of suffering, and even suicidal thoughts [2,4-6]. Social aspects might lead to various types of social loss (loss of social role, status, connection, or job), financial concerns, worries about the family’s future, and dependency [2,4,5]. Moreover, cancer pain seems to lead patients to existential thoughts. It has been associated with spiritual concepts, such as finding meaning, losing faith, fear of uncertainty, and anger toward fate or anger with God [2].

Moreover, cancer pain interferes with the quality of life of patients, and its impact reverberates in the family context [7]. According to the Systematic Transactional Model (STM) [8,9], illnesses encompass a relational dimension and could be viewed as a “*we disease*” since both patients and caregivers share the stress related to pain and coping with it. Specifically, the STM assumes that an interdependence exists between 2 partners in a relationship and postulates that stressors interfere directly or indirectly with both partners in a close relationship [8]. In other words, one partner’s suffering can impact the well-being of another, resulting in the increased suffering of the first partner and so on. This process activates a co-dependence mechanism between patients and caregivers [10] since a family member with a chronic condition needs help from their partner. Still, patients simultaneously feel dependent, perceiving themselves as burdens to their partners [11]. Consequently, caregivers might indirectly perceive an emotional commitment, with feelings of guilt and inability to care for their loved ones, as demonstrated by previous research [7,11,12]. However, even though both patients and their caregivers experience pain, these experiences seem to be represented differently, and little is known about the perspective of caregivers and the patient-caregiver dyad.

Several studies [4,5] on cancer patients emphasized the link between pain and psychoemotional distress, including depressive feelings, anxiety, worries, and fear. The literature mostly focused on the 2 negative states of anxiety and depression in relation to cancer pain [5,13,14]; however, other discrete negative emotions may be activated by pain as well. Sela et al [15] demonstrated that patients with pain tend to mainly experience frustration and exhaustion, followed by anger, helplessness, fear of suffering, and hopelessness. Yet, patients find it difficult to express their emotions, and often some feelings may be overshadowed by others. For example, negative emotions, such as fear, panic, frustration, anxiety, and helplessness, could be hidden by anger [16]. Indeed, anger may be toward cancer, toward those who provide care, or against God, if the patient is a believer [2,16].

A few studies [7,17] have focused on the emotional experiences of caregivers. Sharing the suffering and pain with another person may activate empathetic involvement, making a person more vulnerable to psychological symptoms, including distress, fatigue, and pain. The emotional burden and perceived responsibility of caregivers compromise their ability to care for their loved ones [7,18,19]. Coherently, they seem to be exposed more to feelings of guilt, blame (blaming the pain for the changes caused in the family), anger, or fear (regarding the uncertain future of their loved ones) [20]. Moreover, they may be overwhelmed by feelings of sadness, anxiety, grief, frustration, and helplessness [21].

Despite this evidence and progression in pain management, pain is still underreported and untreated [22-24], representing a major medical unmet need in psycho-oncology [25]. One of the main barriers is patients’ difficulty in reporting pain [26]. This reluctance seems to be related to the lack of knowledge and education about cancer pain. This may result in misbeliefs about pain management. For example, patients may be worried about how to communicate pain, may prioritize curing cancer instead of having relief from pain, or may be convinced that pain is inevitable [26]. A similar challenge is present from the perspective of caregivers, who need adequate education to manage their time and roles, and attend to self-care to reduce the emotional distress related to caregiving [19,27]. Additionally, they need to be provided with problem-solving strategies and to be seen by physicians for their role in pain management [27].

As suggested by Wang et al [28] in a recent systematic review on the unmet needs of cancer patients and their caregivers, qualitative data provide precious insights into the unmet needs of a disease-related experience, such as cancer pain.

Online social groups represent a rich source for qualitative data, as they provide space for users to share their first-hand experiences and receive social support and advice. These platforms have been demonstrated to create a sense of belongingness that helps users (patients and caregivers) feel more understood and less alone, and receive the information needed [29,30]. Moreover, they are useful tools for revealing basic and complex emotions that otherwise are more difficult to capture in traditional settings [31].

### Aims of the Study

This study aimed to capture the whole representation of the cancer pain experience from the perspectives of patients and caregivers. Given that patients directly experience the pain whereas caregivers react to that experience, we were interested in the perspectives of these 2 groups separately.

Specifically, the first aim was to identify the unmet needs of patients and caregivers in relation to cancer pain. Second, this study aimed to detect the reaction to cancer pain in terms of emotions and sentiments by analyzing the textual patterns of both patients and caregivers. Comparisons were made to reveal the difference in reactions to patients’ cancer pain experiences in these 2 groups. 

## Methods

### Data Collection

Data were collected following Pushshift Reddit API Documentation [32] in November 2021. Comments posted on the cancer patient support group on Reddit (with 45,900 subscribers) were sourced from the subreddit [33] using keywords related to cancer pain classification [34,35]: temporal pattern (“acute*pain” and “chronic*pain”), pathophysiology (“somatic*pain,” “visceral*pain,” “neuropathic*pain,” and “nociceptive*pain”), and descriptors of neuropathic pain (“pain*sensation,” “burning*sensation,” “numbness,” “soreness,” “tingling,” “shooting,” “pricking,” and “pins/or needles”). We then manually added common words used by users to refer to pain: “pain,” “hurting,” “aching,” and “discomfort.” All collected posts were screened by the authors CP and MC independently. Duplicate posts were removed, and those unrelated to cancer pain were excluded.

For each post, we collected the following information: data created, number of comments, and username of the poster. Years of posting were determined to convert epochs to human-readable data. The analysis did not consider any reference to names or people mentioned in the posts to respect the anonymity of users.

### Ethical Considerations

Ethical committee approval was not requested since data collection and analyses involved public online materials.

### Statistical Analysis

#### Descriptive Statistics and Word Frequency

This study involved a manual categorization procedure whereby posts were read and assigned to pre-existing categories. Two reviewers (authors CF and MC) created these pre-existing categories based on 100 posts that were randomly generated by Google’s random generator.

Guided by the preliminary codes, the 2 reviewers categorized all posts (n=783). When preliminary codes did not match the content of posts, new codes were added to the broad categories after agreement between the 2 coders. Conflicting codes were solved through discussion to generate the final list. Both reviewers coded all posts.

Quantitative data analyses were performed using RStudio v.2022.02.3 [36]. Interrater reliability was assessed by calculating the Cohen kappa, with values of <0 indicating no agreement, 0.01-0.20 indicating slight agreement, 0.21-0.40 indicating fair agreement, 0.41-0.60 indicating moderate agreement, 0.61-0.80 indicating substantial agreement, and 0.81-1 indicating almost perfect agreement [37].

Text mining was performed to clear the data and compare how patients and caregivers describe their experiences in relation to cancer pain, and a word cloud (ie, a method to identify the most frequently used words in text) was generated with the “tm” [38] and “wordcloud” [39] packages in R.

#### Emotion and Sentiment Analysis

Emotion and sentiment analysis was performed on the posts of patients and caregivers with the “syuzhet” R package [40]. We considered 8 basic emotions (anger, fear, anticipation, trust, surprise, sadness, joy, and disgust) and 2 sentiments (positive and negative) based on the NRC Emotion Lexicon [41,42]. The lexicon allows for determining the emotions and sentiments associated with each word. The association between the target word and the emotion was indicated with either 0 (no association) or 1 (association present). ﻿Each term can be related to more than one emotion and have a positive, negative, or polarity orientation. Negative words are mostly associated with anger, fear, disgust, and sadness, whereas positive words are primarily associated with anticipation, joy, and trust. Surprise may be categorized with positive or negative emotions and sentiments depending on the target words.

We followed a series of steps to assess the distribution of our data. First, we used the R software to select relevant posts and “unnest” the text. This involved breaking down the text into individual sentence units. Consequently, we obtained a total of 5577 sentences for patients and 2052 sentences for caregivers. Each sentence was then treated as a separate data point within the R software. Next, we applied the emotion analysis to this data set of sentences using the “get_nrc_sentiment” function. This analysis produced a new data set with rows representing individual sentences and columns representing different emotions. The association between a sentence and an emotion was established when one or more words within the sentence matched that emotion. Hence, each sentence in the resulting table was assigned a numerical value for each emotion, indicating the emotional intensity of that sentence. Subsequently, we organized the data frame in this format to examine whether the distribution of emotions across the sentences (data points) followed a normal distribution. To achieve this, we employed the Shapiro test, a statistical test used to assess the conformity of data to the assumption of normal distribution. Our results indicated that the *P* value obtained from the Shapiro test was less than the predetermined significance level (.05). Therefore, we concluded that the distribution of emotions across the sentences did not adhere to the normal distribution assumption.

Since the data did not follow a normal distribution, we proceeded to perform the Wilcoxon rank sum test. This test is a nonparametric alternative when the normality assumption is not confirmed. The test performs well with unequal sample sizes as well [43].

#### Hierarchical Clustering

Hierarchical clustering was performed on comments from caregivers and patients, using the “dendexten” R package [44]. Hierarchical clustering is a k-means–based method used to identify clusters in a data set. This technique groups observations into clusters without a prespecified number of sets and creates a tree-based representation of observations called a dendrogram. We used the agglomerative clustering method AGNES (Agglomerative Nesting), which follows a bottom-up approach and considers each data point as a separate cluster. It iteratively merges the most similar clusters based on a distance metric until a stopping criterion, such as a predetermined number of clusters, is reached. The resulting dendrogram provides a hierarchical representation of the clusters that can be used to determine the optimal number of clusters.

Moreover, the agglomerative coefficient (ie, the amount of clustering structure found) was calculated. A coefficient closer to 1 is considered to indicate a strong clustering structure. The Ward method, which minimizes the total within-cluster variance, was used to create the cluster dendrogram.

Finally, dendrograms were compared using the function “tanglegram,” which plots 2 dendrograms side by side with their labels connected with lines. The alignment quality was calculated with the function “entanglement” to determine the optimal number of clusters and the validity of the results. A good alignment is guaranteed with a lower entanglement coefficient (ranging from 1 [whole entanglement] to 0 [no entanglement]).

The labels of each cluster were attributed after agreement between the authors CF and MC. For details, see the guidelines provided by Galili [44] and Kassambara [45].

Finally, we interpreted the product clusters and labeled them depending on the hierarchical clustering performed, considering the context from which the words come.

## Results

### Descriptive Statistics and Word Frequency

Interrater reliability for manual coding indicated perfect agreement (from 0.98 to 1) for all broad categories and codes (Table 1).

A total of 783 public comments between April 2011 and November 2021 were identified. Of the 783 comments, 679 (161 from caregivers and 518 from patients) were included in the final database since our aim was to focus on the perspectives of patients and caregivers. Therefore, 104 posts were excluded from the analysis since the user type was unknown (n=93) or there was a referral to a health care professional (n=11). Details are provided in Multimedia Appendix 1.

**Table 1:** Broad categories, related codes, and interrater reliability results.

Broad categories and codes		Interrater reliability (n=783)	*P* value
**Pain dimension**		0.99	<.001
	Physical		
	Psychological		
	Both^a^		
**Type of comment**		0.98	<.001
	Advice		
	Experience		
	Both^b^		
	Question		
**Type of user**		1.00	<.001
	Patient		
	Caregiver		
	HC^c^		
	Unknown		
**Type of pain**		0.99	<.001
	Acute		
	Chronic		
	Acute neuropathy		
	Chronic neuropathy		
	Neuropathy		
	Somatic		
	Visceral		
	Unknown		
**Type of cancer^d^**		0.98	<.001
	Blood^e^		
	Breast		
	Gynecological^f^		
	Pancreatic		
	Melanoma		
	Sarcoma		
	Lung		
	Colorectal		
	Brain		
	Others		
	Not diagnosed		
	NA^g^		

^a^Physical and psychological.

^b^Advice and experience.

^c^HC: health care professional.

^d^Type of cancer of patients discussed in the posts.

^e^Leukemia, lymphoma, and myeloma.

^f^Ovarian, cervical, uterine, vaginal, and vulvar.

^g^NA: not available.

#### Patients’ Comments

Among the 679 comments included, 518 (76.3%) were posted by patients. Regarding cancer pain, the most frequent dimension was the physical dimension (359/518, 69.3%). In comparison, 23.7% (123/518) of the comments were focused on both dimensions of pain (physical and psychological), and only 7.1% (37/518) were focused on the psychological dimension.

Among the 518 comments, 219 (42.3%) did not specify the type of pain. Among the comments that did specify the type of pain, the most frequent type was neuropathy (95/518, 18.3%), followed by chronic (80/518, 15.4%), acute (51/518, 9.8%), somatic (2/518, 0.4%), and visceral (1/518, 0.2%) pain. Regarding neuropathy, we found that 10.0% (52/518) of posts involved chronic neuropathy, while 3.5% (18/518) involved acute neuropathy.

In most posts (422/518, 81.5%), patients shared their first-hand experiences and provided information to others in a similar condition. A smaller portion of posts (53/518, 10.2%) provided advice, and some posts (9/518, 1.7%) posed a question. Further details are provided in Multimedia Appendix 1. Table 2 shows the top 35 most used words and their frequencies.

**Table 2:** The top 35 words regarding cancer pain most frequently used by patients and caregivers.

Number	Patients (N=87,136)		Caregivers (N=33,583)	
	Word^a^	Value, n	Word^a^	Value, n
1	Pain	615	Pain	217
2	Feel	405	Cancer	196
3	Cancer	384	Time	159
4	Day	335	Can	148
5	Can	328	Feel	138
6	Time	300	Help	107
7	Treatment	260	Want	101
8	Week	232	Mom	96
9	Help	230	Know	96
10	Chemotherapy	223	Day	93
11	Year	218	Dad	90
12	Back	189	Doctor	78
13	Know	189	Week	74
14	Take	189	Thing	72
15	Now	183	Think	71
16	Say	170	Treatment	70
17	Month	169	Now	68
18	Surgery	164	Hospital	64
19	Side	158	Take	63
20	Life	158	Sorry	62
21	Doctor	152	Chemotherapy	62
22	Lot	146	Need	62
23	Good	142	Back	59
24	Start	138	Family	59
25	Try	136	Lot	58
26	Work	136	People	58
27	Need	136	Hope	56
28	Effect	135	Last	54
29	Think	133	Love	54
30	Soreness	132	Month	54
31	Hurt	130	Life	52
32	Cause	129	Try	51
33	Radiation	129	Care	50
34	Use	127	Work	50
35	Thing	123	Way	50

^a^Common words: pain, can, cancer, chemotherapy, day, doctor, feel, help, know, lot, need, now, thing, think, time, week, back, life, month, take, treatment, try, and work.

#### Caregivers’ Comments

Among the 679 comments included, 161 (23.7%) were posted by caregivers. Most caregiver posts discussed pain, focusing on the psychological and physical dimensions (67/161, 41.6%). Some focused only on the physical dimension (50/161, 31.1%), and others focused only on the psychological dimension (44/161, 27.3%).

Among the 161 comments, 123 (76.4%) did not specify the type of pain. Among the comments that did specify the type of pain, the most frequent type was chronic pain (18/161, 11.2%), followed by neuropathy (12/161, 7.5%) and acute pain (4/161, 2.5%). Somatic and visceral pain was not found in their comments. Regarding neuropathy, we found that only 2.5% (4/161) of posts involved chronic neuropathy, while 0.6% (1/161) involved acute neuropathy.

In general, caregivers mostly shared the experience of their loved ones with cancer in their posts (130/161, 80.7%). Caregivers shared their experience as well as provided information in 11.2% (18/161) of posts, and they only provided advice in 8.1% (13/161) of posts. Further details are provided in Multimedia Appendix 1. Table 2 shows the top 35 most used words and their frequencies.

#### Word Cloud Comparison

A word cloud comparing patients’ and caregivers’ words when discussing the cancer pain experience is presented in Multimedia Appendix 2.

The word cloud was created by analyzing the most frequent words. Patients more frequently used terms describing the physical aspects of pain (“neuropathy,” “sensation,” “nerve,” “hurt,” “fatigue,” etc), causes of pain related to treatment (including “radiation,” “surgery,” and “chemotherapy”) or a specific procedure to detect cancer (“biopsy” and “scan”), aspects related to pharmacological treatments taken for managing pain (“drug,” “oxaliplatin,” “dose,” and “addiction”), and their related side effects (“nausea,” “soreness,” “hair” loss, “scar,” “numbness,” “cold” sensation, etc) compared with caregivers. The only psychological terms used frequently in relation to pain were “worry” and “scary.”

On the other hand, caregivers used words related to psychosocial aspects of pain (“family,” including “dad,” “mom,” “wife,” etc; “sorry,” “help,” “memories,” “care,” “doctor,” “death,” “understand,” “remember,” etc) more frequently compared with patients. In their case, the physical aspects or related side effects of pain and treatments were described less frequently (eg, “morphine,” “stage,” and “acute”).

### Emotion and Sentiment Analysis

#### Patients’ Comments

Multimedia Appendix 3 displays 8 emotions (anger, fear, anticipation, trust, surprise, sadness, joy, and disgust) and 2 sentiments (positive and negative) associated with the target words used by patients when discussing cancer pain. The total number of patients’ words was 87,136, and the total number of sentences extracted was 5577.

Based on the associations with target words, the negative sentiment (mean=0.83) was higher than the positive one (mean=0.58), with sadness (mean=0.57) and fear (mean=0.56) being the most prevalent negative emotions, followed by anger (mean=0.30) and disgust (mean=0.26). On the other hand, trust (mean=0.40) and anticipation (mean=0.35) were the most pervasive positive emotions, followed by joy (mean=0.25). The least prevalent emotion was surprise (mean=0.17).

The following extracted sentences (from post IDs P2 and P258) well exemplify these sentiments and emotions:

- Sentences extracted from post ID P2

The worst thing about cancer is the fear, and the fear is driven by lack of knowledge.

The more you know about your situation -- and the treatment options, and the side effects, and the medical team, and the support services -- the easier it is to handle it.

I'm not saying it becomes easy, full-stop, but it does make it easier.

Knowledge is power, it pushes back the darkness.

And that goes for the people you love, too, the ones you're trying to spare from pain and worry.

If they don't know what's going on, they'll worry more.

Giving yourself and them, information will make things less opaque and scary.

Having a skilled team of medical experts and a support system will, too.

Finally, it is TOTALLY NATURAL to feel the way you're feeling!

And as always, #FUCKCANCER.

- Sentences extracted from post ID P258

My cancer returned when I was 22, my leg was amputated a week later.

I had an endless supply of drugs to deal with the pain, both” real” and phantom limb pains.

I’ve felt a lot of the things you currently feel.

I hate feeling weak, and I hate relying on people around me.

Some days is worse than others, but I have something I can look back on and use as a reference that it can get better.

I initially got the cancer diagnosis when I was 14, after 5 years of unexplainable pain. I did chemo for 2 years, radiation therapy for 6 weeks, and 6 surgeries in total.

One of these surgeries involved temporarily cutting off the blood supply between my leg and the rest of my body, pumping my leg with extremely toxic chemo that took my leg to 47C (or 117F) degrees.

I was a kid when I lost everything.

I experienced insane amounts of pain between 14-17.

But after treatment, my foot was still broken, and I suffered from osteoporosis in my lower leg; I was shattering bones in my foot just from walking.

I’ve had chronic pain for 14 years and I’m 24 years old.

I can vividly remember all of the times I nearly died.

I remember bleeding in my mouth from eating, waking up in the middle of the night screaming in pain from the full-body cramps, the painful wound on my foot from the radiation therapy, and a seemingly endless list of side effects.

#### Caregivers’ Comments

Multimedia Appendix 4 displays 8 emotions (anger, fear, anticipation, trust, surprise, sadness, joy, and disgust) and 2 sentiments (positive and negative) associated with the target words used by caregivers when discussing cancer pain. The total number of caregivers’ words was 33,583, and the total number of sentences extracted was 2052.

Based on the associations with target words, the negative sentiment (mean=0.78) was higher than the positive one (mean=0.64), with sadness (mean=0.61) and fear (mean=0.55) being the most prevalent negative emotions, followed by anger (mean=0.31) and disgust (mean=0.25). On the other hand, trust (mean=0.45) and anticipation (mean=0.38) were the most pervasive positive emotions, followed by joy (mean=0.28). The least prevalent emotion was surprise (mean=0.16).

The following extracted sentences (from post IDs C717 and C100) well exemplify these sentiments and emotions:

- Sentences extracted from post ID C717

My gf has stage IV lung cancer, and I cried a few times (I haven't cried for several years before that) but I feel like I am mostly in a “functioning” mode that keeps me going, but I am absolutely over the top overwhelmed with emotions and thoughts, but I know I am no good for my gf either if I just give up.

With long times of sickness and going through all that with someone, some people even feel relieved when their loved ones die and feel very guilty, but I think in most cases it is a relief that their loved one don´t have to suffer anymore, I didn´t cry when my dad died after months of being in and out of the hospital and intensive care, but it hit me later.

- Sentences extracted from post ID C100

I lost my husband 47 days ago (this is day 48), and as devastatingly painful as it was to lose him after 24 years together, every time I: 1) remember his struggles in the two months prior to losing him; 2) remember all the times he said he didn't want to be sicker from the treatment from the disease; 3) look at pictures and videos from his final days; it helps me accept that he is gone.

I absolutely loathe the “he's no longer in pain” sentiment, but I've realized what I actually miss most of all are the times before he got sick.

Truth be told, his last two months were increasingly terrible with every passing day.

I can't tell you how many times he said to me, «this is not living».

I share this in case it helps.

If your mom is not yet hospitalized and can take care of her own needs, there is still hope for her.

In my husband's case, that hope evaporated early, though, and if and when it begins evaporating for your mom, the best thing you can do is remind yourself that « keeping her alive » doesn't mean she's actually «living».

That could help you let her go.

#### Wilcoxon Rank Sum Test for the Emotional Scores of Patients and Caregivers

The Wilcoxon rank sum test was used to analyze the differences in emotion and sentiment scores between patients and caregivers.

The test showed that patients expressed a negative sentiment more often than caregivers (mean_P_rank_=3845.24 vs mean_C_rank_=3732.81; z=−2.14; *P*<.001), whereas caregivers expressed a positive sentiment more often than patients (mean_P_rank_=3784.53 vs mean_C_rank_=3897.81; z=−2.26; *P*<.001), with trust (mean_P_rank_=3763.79 vs mean_C_rank_=3954.18; z=−4.12; *P*<.001) and joy (mean_P_rank_=3792.90 vs mean_C_rank_=3875.06; z=−2.03; *P*<.001) being the most prevalent positive emotions. Details are provided in Table 3.

**Table 3:** Wilcoxon rank sum test results for emotion and sentiment scores between patients and caregivers.

Emotion	Caregiver mean_rank_ (n=2052)	Patient mean_rank_ (n=5577)	*U*	z	*P* value
Anger	3843.45	3804.53	5663619.00	−0.908	.36
Disgust	3782.24	3827.06	5654769.50	−1.09	.27
Fear	3810.52	3816.65	5712817.00	−0.12	.90
Sadness	3868.65	3795.26	5611917.00	−1.47	.14
Anticipation	3862.46	3797.54	5624621.50	−1.45	.15
Joy	3875.06	3792.90	5598766.00	−2.03	<.001
Surprise	3821.11	3812.75	5709462.50	−0.24	.81
Trust	3954.18	3763.79	5436407.50	−4.12	<.001
Negative^a^	3732.81	3845.24	5553344.50	−2.14	<.001
Positive^a^	3897.81	3784.53	5552077.00	−2.26	<.001

^a^A target word may be associated with one or more emotions and 1 of the 2 polarities (negative or positive). While a target word is always associated with 1 of the 2 polarities, it is not always associated with a specific emotion.

### Hierarchical Clustering

Multimedia Appendix 5 shows the hierarchical clustering findings. The optimal number of clusters for patients was 2, which belonged to 1 root representing patients’ pain perspective. The agglomerative coefficient with the Ward method was 0.72, which demonstrated a solid clustering structure. Similarly, in the hierarchical clustering of caregivers, the optimal number of clusters was 2, which belonged to 1 root representing caregivers’ pain perspective. In this case, the agglomerative coefficient was 0.80.

**Figure 1:**
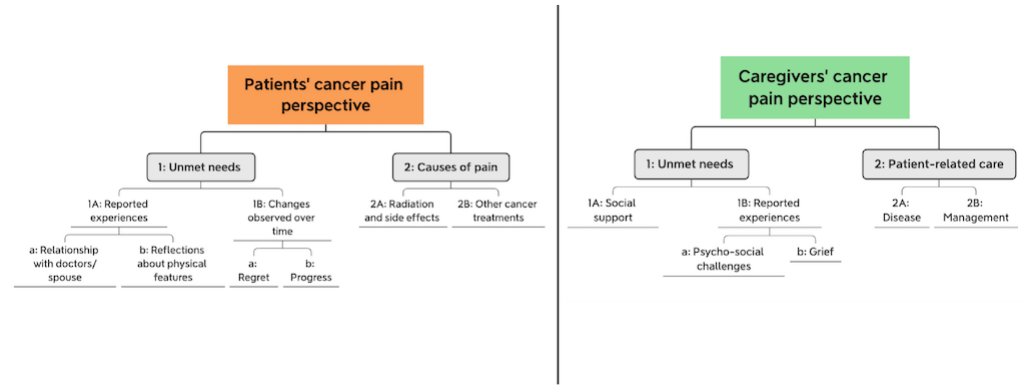
Patients’ and caregivers’ product clusters with labels.

Figure 1 shows the labels applied to interpret the product clusters.

In the case of patients, the 2 main clusters were labeled as (1) *unmet needs* and (2) *cause of pain*. This first cluster of *unmet needs* included 2 nodes named (1A) *reported experiences*, with the subclusters (a) *relationship with doctors/spouse* and (b) *reflections on physical features*; and (1B) *changes observed over time*, with the subclusters (a) *regret* and (b) *progress*. The second cluster of *cause of pain* included 2 nodes named (2A) *radiation and side effects* and (2B) *other* *cancer treatments*.

Regarding patients’ reported experiences (cluster 1A), the relationship with the spouse (subcluster a of cluster 1A) is well represented in the following post (post ID P478):

I got diagnosed about 5 weeks ago with stage IV. It has completely changed the relationship…From my end, I now see my spouse as a caregiver instead of a spouse. I feel horrible about it and try and remind myself that he is my sexy husband who I adore, but when he is wiping my butt and stuff, it's hard to remember that. Sometimes I see him and just cry because I want to see him as my sexy husband, but it just seems impossible right now.

The relationship with doctors (subcluster a of cluster 1A) was related to the need of reassurance. This is evident in the following post (post ID P399):

I know how bone cancer feels and how recovery feels.

This is cancer. But everyone thinks I'm just “imagining” it because I'm afraid of it returning. But I truly know I'm not. I know my body.

I'm just waiting for my doctor to tell me so I can get on with treatment.

Regarding physical features (subcluster b of cluster 1A), patients shared the impact of pain on different parts of their bodies. Moreover, they described the physical symptoms (eg, numbness and soreness) experienced.

Had Stage 0 breast cancer (DCIS) and a lumpectomy with a scar on the side of my breast, but close to the armpit. Has been a year and it was slightly painful/sensitive for many months afterward. Even now I still get some pain where the scar tissue is (burning and/or aching). From what I have read in blogs/chatrooms online, this is normal and can last for many years .... Glad to hear you do NOT have cancer. :-)

Post ID P429

Concerning the changes observed over time (cluster 1B), patients reported regret (subcluster a of cluster 1B) about the action taken during recovery because of the difficulty of waiting and wish to have quick progress (subcluster b of cluster 1B) regarding the right time and the need to be as they were before the diagnosis.

I’m hoping to move on to using a stationary bike soon but like you said I have to take it easy. I’ve been pushing myself and regretting it afterwards. Progress is never fast enough.

Post ID P127

As for caregivers, the first large cluster was labeled (1) *unmet needs.* This cluster included 2 nodes named (1A) *social support* and (1B) *reported experiences*, with the subclusters (a) *psychosocial challenges* and (b) *grief*. The second cluster was labeled (2) *patient-related* *care*. This cluster included 2 nodes named (2A) *disease* and (2B) *management.*

Regarding social support (cluster 1A), caregivers expressed this need due to the responsibility they felt in relation to making a choice for the patients. For example, a caregiver wrote:

I would do ask for support and make his time as comfortable as possible. It is not your fault and you need to remember that. It is no ones fault. I do hope he is comfortable, and whatever choice you make, I’m sure will be the right one.

Post ID C128

Regarding their experiences (cluster 1B), caregivers also shared the need to be understood for the burden related to the psychosocial challenges (subcluster a of cluster 1B) of pain management, the care of their loved ones, and the grief related to losing someone (subcluster b of cluster 1B).

The following extracted posts are some examples of what caregivers shared on the platform with other caregivers:

I’m so terribly sorry for your loss. I lost my mom to cancer in August too. I wish I could say it gets easier but I found it comes to you in waves. One second you’ll be fine and the next second you’ll be crying. Followed by numbness. It’s hard to watch them slowly fade away from us. And there’s nothing we could’ve done to help save them. It’s hard. Reach out for help with extended family to see if they can help take some of the burden off of you. If you ever need someone to talk to you can always shoot me a message here.

Post ID C697

…Not many people can verbalize what I am feeling…Our pains and struggles are different but hauntingly similar…. Often people don’t and cannot understand. Even worse, they often don’t *want* to understand, especially when you’re young….My grief and suffering make people uncomfortable. My husband’s suffering and mortality make them uncomfortable…They don’t want to see it, so they only see what they want to see. They see a young guy that looks good for having cancer. They dismiss his deficits as “well sometimes I forget things, get lost, or have brain farts! Totally normal!” This isn’t a brain fart or a slight delay in finding words. This isn’t a “shit I forgot to bring my lunch today.” It’s much deeper and more consistent than that. This isn’t normal…

Post ID C356

Additionally, the comparison between the 2 dendrograms demonstrated good entanglement (entanglement coefficient=0.28), with only partial similarity regarding the clade consisting of the 2 words “help” and “may” in both the patient and caregiver dendrograms. Based on the dictionary of Loughran and Mcdonald [46], both words represent uncertainty. Thereby, we labeled the textual node shared by both dendrograms as *uncertainty*. See Multimedia Appendix 5 for more details.

For example, patient #340 reported uncertainty (*what if*) related to the disease condition and unpredictable future. This uncertainty can bring worries and fears.

What I came to realize (with the help of therapy) is, that there are a lot of What ifs attached to cancer and the anxiety that comes with it. What if my cancer spreads?, What if it won't go away?, What if it comes back?, What if they find something on my next scan?, or my personal favorite: What if they overlook something on my next scan?. But for every What if we will worry about, there is an infinite number of What ifs we don't even think about. We can't predict the future or how it will develop but if we worry what bad could happen, we might miss the good that can happen too. Or frankly speaking if it's a beautiful sunny day outside, I won't run around with an open umbrella because it might start raining or a I could get shit on by a bird.

As for caregivers, uncertainty was related to the grief and fear of losing a loved one. For example, caregiver #159 expressed these feelings by supporting another caregiver.

Your story caught my attention immediately. I know the pain, fear and the uncertainty you are going through. You see, my daughter died 3 weeks ago after a 3 1/2 year battle with leukemia. She was 12 years and 5 months old to the day. I will elaborate some, not to compound anything you are going through, but to let you know, you are not alone.

## Discussion

### General Overview of the Findings

This study aimed to investigate the whole representation of cancer pain, considering the perspective of patients and caregivers. Overall, we found that patients and caregivers shared 3 types of content on the Reddit cancer social group about pain: experience, advice, and questions. The most frequent type of shared content in both groups was experience, followed by advice. The questions asked were only by patients. Moreover, different types of pain were covered in the narratives of both users. The most frequent types of pain discussed were neuropathy, chronic pain, and acute pain.

The language used to describe the experiences differed in these 2 groups. This was expected since the experiences of patients and caregivers differ regarding cancer pain. While patients have first-hand experience of pain, the experience of caregivers refers to their reaction to it. This said, patients described pain focusing more on the physical dimension (“neuropathy,” “sensation,” “chronic,” “fatigue,” etc), causes of pain related to treatment (“radiation,” “surgery,” and “chemotherapy”) or a specific procedure to detect cancer (“biopsy” and “scan”), side effects of treatment (“nausea,” “soreness,” “hair” loss, “scar,” “numbness,” “cold” sensation, etc), and aspects related to pharmacological treatments taken for managing pain (“drug,” “oxaliplatin,” “dose,” etc). As for caregivers, they described the experience in terms of the reaction to the suffering of their care and the impact that the experience had in their life, discussing the psychosocial aspects when confronting other caregivers (“family,” “sorry,” “help,” “memories,” “grief,” etc). Coherently, regarding patients’ posts, 69.3% (359/518) covered the physical aspects of pain solely, whereas only 31.1% (50/161) of caregivers’ posts discussed them. As for the psychological dimension of the pain experience, 27.3% (44/161) of caregivers’ posts covered this dimension, whereas this percentage was only 7.1% (37/518) for patients.

According to the STM, each disease may be viewed as a “we disease,” affecting patients and their family members. This process results from the interdependence between the 2 actors [8]. Specifically, patients often depend on their caregivers [11] when they have a chronic condition. This may be due to patients’ loss of autonomy and functionality that can contribute to creating a co-dependence mechanism of the patients on their partners [10]. Such an increased need for care may result in a perceived burden on the family members [47,48]. For instance, this sense of burden was well represented in one of the posts from our data. A caregiver who shared their experience was providing another caregiver with support and understanding:

As for those witnessing his pain: I'm sorry, I am so sorry … My only advice is to take turns. Everyone experiencing this needs some distance from it from time to time…. If I don't spend some time away from the pain, I will lose my mind.

Post ID C261

While being a heavy burden, the way in which caregivers deal and cope with caregiving can also be affected by the quality of their relationship with the patient. The closeness between the partners, the time spent together, and the general strength of their relationship may impact multiple aspects of both patients’ and caregivers’ experiences. As the STM suggests [8], the caregiver’s resources may expand the patient’s resources, creating new synergies for fighting against pain. This is evident when there is a strong bond present between them. For instance, a caregiver wrote:

One could certainly have that reaction of hating every bit of the lifestyle change, and perhaps at times it may seem just 100% detestable and harrowing, but as is the case with any event that occurs in life, a significant amount depends on how you participate in it and how you experience it.

I am 32 years old and my husband was 31 when he passed away in early March. We were together for over ten years and got married just before he passed away. My one advice to you is, be the hero you can be. Our job isn’t to treat their cancer, medically that is. That’s the job of the doctors, rightfully so. Instead, focus on doing what you are capable of doing, which is being her partner, being her companion through this new life

…

So in a way, I wanted him to think that it was both of us who got diagnosed. He had to bear the brunt of it obviously... but no one can survive cancer alone. […]. I was there to listen to him and empathize with him as he expressed the different types of pain he was feeling. We both knew I couldn’t cure his symptoms, but I did what I could— […].Reading through some of his notes he left behind, I realized I did the right thing. I was so relieved when I read how much it meant to him that I was there for him.

Post ID C376

Even if the literature has recently focused more on caregivers’ experiences as well [49,50], their unmet needs and implicit emotional side of cancer pain are still underrepresented. This work considers this gap and the importance of the mutual influence between patients and caregivers. 

### How Patients and Caregivers Live With Cancer Pain: Unmet Needs

A person’s significant need that is not fulfilled is referred to as an unmet need [51]. Our first aim was to reveal the critical concerns related to cancer pain expressed by patients and caregivers in their posts and the specific needs related to that experience.

Regarding patients, the hierarchical cluster analysis showed that their pain-related primary needs were *relationships with the doctors/spouse* (ie, seeking the reassurance/opinion of the physician about pain, and seeing the spouse primarily as a caregiver instead of a spouse) and *reflections on the physical features of pain* (eg, location of pain in the body; chronic pain; stage of cancer; and body sensations such as numbness, soreness, etc*).* Therefore, patients emphasized the physical aspects of pain. Several reasons may explain this narrowed focus.

The first and most obvious reason is that the pain experienced by patients involves physical aspects. It is related to tissue damage from oncological treatments, surgery, or cancer itself [35]. This aligns with cluster 2 of patients’ words (*causes of pain*), in which patients mainly discussed *radiation and its related side effects* and *other cancer treatments* (eg, chemotherapy) as the primary causes of pain. Consequently, it may be intuitive for patients to assume that having physical roots, pain would only have physical outcomes.

However, the physical sensation of pain is not the only reason for patients to mostly speak about pain in terms of physical symptoms. Patients are generally directed by their physicians to focus on their physical characteristics during consultation sessions [35,52], with questions such as “What was your pain intensity in the last 24 hours?” This may contribute to their tendency to become aware of their physical experiences rather than their psychological ones. Moreover, the typical response of a medical health care provider to a pain complaint is the prescription of a pharmacological treatment (painkiller) according to the World Health Organization’s analgesic ladder [53]. Overall, doctors may implicitly convey to patients that pain is only a biological concern by attempting only physical relief. For instance, this issue was well described in the following extracted sentence of a patient:

I just kept adjusting and moving in my seat. My doctor said it was probably the normal side effect of bone pain, but I never thought the pain was that bad until he prescribed me some painkillers, and I fully relaxed and could sit still.

Post ID P1

However, interestingly, when patients retrospectively reflected on their pain (cluster of patients’ words called *changes observed over time*), they tended to go further. They also shared their psychological needs, such as the desire to return to their old self before the pain, influencing them to push themselves to be more active. This led them to *regret* the action taken and realize that *progress* is never fast enough.

As for caregivers, the primary needs discussed were the *psychosocial challenges* that they experience because of the condition of their loved ones (eg, economic and work issues, reflections on the time passed, worsening of the disease, wishing for a better prognosis, hope that the treatment will work, etc) and *grief* (eg, feeling numb after a death, self-blame, loss, hope for their loved ones, etc).

Grief is a familiar feeling experienced by caregivers, and if not well managed, it could remain after 6 months to 1 year following the loss of their loved ones [54]. However, as also seen in the example above, grief is experienced not only as a response to the death of loved ones but also as a response to the idea of losing them. The caregiver’s suffering could be caused by the caregiving itself (when it becomes a burden), or by the grief about losing or the idea of losing a loved one. As recognized by Allen et al [54], it is fundamental to take care of the suffering experienced by caregivers by identifying those more at risk in order to target interventions for them.

Thereby, caregivers mainly focused on the psychological dimension of pain rather than the physical dimension (which they only mentioned when discussing the patient’s care; see cluster 2 of caregivers’ words).

The reasons for this may be multiple. First and most obvious, they are not experiencing cancer pain in the first person, and they live these experiences through the lens of a caregiver. Second, their primary role is to provide care and support to reduce the impact of pain in the patients. However, when this goal is not fully achieved, family members may experience anger, helplessness, powerlessness, exhaustion, spiritual distress, lack of confidence, self-blame, and burden from caring [20,27]. All of these represent the psychological challenges that caregivers face every day living with the suffering of their loved ones (presented in the cluster of *psychological challenges*). Third, to avoid getting overwhelmed, caregivers may need to seek support from others and create a sense of community as represented in the cluster of *social support*. Social support has been demonstrated to be one of the most critical unmet needs for caregivers and patients [28], which could reduce pain perception in cancer patients [5,55] and mitigate emotional distress in caregivers [27,56]. Still, it is fundamental to see the patients’ and caregivers’ needs in the overall well-being of the patient-caregiver dyad rather than just that of the patients [57], as the STM [8] explains.

Despite caregivers and patients having different concerns and expressing different needs, we found that they share a common theme. This theme is represented by *uncertainty*. Uncertainty is a familiar feeling among patients with cancer pain [28,58,59]. As suggested by the theory of uncertainty [60], it comes when the illness is unpredictable, the prognosis is bad, the disease is still progressing, and symptoms worsen. For patients, uncertainty is expressed as “*what if*” in relation to the condition’s progression and their future, as reported above. It has been demonstrated that cancer patients with pain compared to those without pain showed a higher level of uncertainty. In these patients with pain, uncertainty predicted a lower level of hope [58].

Moreover, it may lead the patients to lose control of the situation and may worsen their pain management [59]. As for caregivers, the uncertainty is often in relation to their loved ones. For them, uncertainty may lead to anticipating grief and may consequently increase the burden of caregiving [61].

As shown in a recent systematic review [62], uncertainty management interventions are composed of a wide array of components in which information support has a key role in managing uncertainty. Lack of education is one of the most prevalent barriers to pain management. This is true for not only patients and their caregivers, but also health care providers who still have misconceptions about morphine and pain treatment (eg, painkillers will lead to addiction, cancer pain is inevitable and patients cannot fully achieve relief through therapy, etc) [63]. The focus on pain management is crucial as it can inform the health care professionals who tailor interventions for patients and their caregivers faced with uncertainty. Such interventions are especially needed for those experiencing chronic pain. eHealth tools may represent a possible option for such interventions [64-66].

### Emotional Narratives of Patients and Caregivers

In this study, our second aim was to detect the emotional activation associated with cancer pain in the textual patterns of both patients and caregivers. It is worth noting that there existed a disparity in the number of posts made by patients and caregivers, with patients’ posts being twice as many as those made by caregivers. Thus, in the subsequent sections pertaining to the outcomes, we compared the emotional proportions and distributions between the 2 groups. The comparison was not intended to be numerical but rather proportional, focusing on the emotional distribution between patient and caregiver posts.

Overall, we found that the emotional activation in both users’ narratives was high. Compared with caregivers, the negative sentiment expressed by patients was significantly higher. Contrarily, caregivers more frequently expressed positive feelings than patients. The prevalence of negative sentiments among patients with cancer is in line with other findings in the literature [4,5,15]. Still, to our knowledge, no studies have focused on patients’ and caregivers’ reactions to pain in terms of emotions and sentiments expressed.

The differences in positive and negative sentiments in our groups may be due to the divergent experiences and the roles that patients and caregivers adopt to cope with pain. Patients live the experience of pain in the first person. They may have to confront the difficulty of managing their disease over time from diagnosis to long-term survivorship (eg, receiving treatments and facing their side effects). Therefore, they focus more on the negative aspects. On the other hand, caregivers often have to adopt the role of a supporter, who maintains an optimistic mindset for both of them, and they sometimes underestimate the difficulties that may emerge on the medical pathway. Indeed, our data showed that caregivers expressed significantly higher trust and joy among positive emotions than patients, representing an optimistic outlook.

As for the specific emotions (anger, disgust, fear, and sadness) primarily associated with negative sentiment, we did not find significant differences between the 2 groups, with a homogeneity in terms of the negative emotions felt. Looking at each group separately, the 2 most frequently expressed negative emotions were sadness and fear in patients’ and caregivers’ narratives. This finding aligns with the fear-avoidance model [67,68], which assumes fear and avoidant behaviors as the primary mechanisms of the experience of pain, activating feelings of depression and disability. Our results stress that the first-hand experience of pain by patients and the third-hand experience of pain by caregivers elicit the same negative emotions outlined in the fear-avoidance model (fear and sadness). However, both groups may express these emotions for 2 different reasons. Specifically, patients may be scared of cancer reoccurrence [69,70], and the misconception of pain may elicit this fear as a sign of failure of treatment or disease progression [71]. On the other hand, caregivers may blame themselves for being incapable of caregiving [72] or feel fear and uncertainty for the future of their loved ones [20].

### Limitations

Our study has some limitations. First, given that the data were retrieved from an online social network, demographics and user personal characteristics (eg, personality, anxiety, depression, etc) were missing from our analyses.

As for interpersonal characteristics, we could not match patients to their caregivers. The source of the data (the cancer subreddit) did not provide such information. Even though some descriptors of the relationship were described in some comments (eg, time spent together), the number of such comments was too small for creating separate variables for relationship characteristics.

Another consideration we would like to make is regarding the users’ cultural backgrounds. Reddit users are mostly based in the United States, followed by the United Kingdom and Canada [73]. This should be considered when interpreting and generalizing the results from our work. Cultural background plays an important role in patients’ and caregivers’ expressions and experiences regarding pain. Therefore, some of the findings outlined in this work may not apply to people with different cultural backgrounds.

Moreover, it is important to note that we used word clouds as a descriptive analysis in this study. While word clouds can provide a visual summary of frequently mentioned words, they may not fully capture the nuanced nature of individuals’ experiences or account for contextual factors and connotations associated with specific words. We would like to emphasize the need for caution in interpreting word clouds, as they may oversimplify or misunderstand the intricacies of the data. By discussing these limitations, we aim to provide a more balanced understanding of the strengths and weaknesses of word clouds. Finally, information about cancer type and treatment type was lacking (variables that may have specific implications for the patients’ pain). Therefore, we could not consider these variables in our analyses. This may impact the generalizability of the findings.

Overall, the lack of participant characteristics represents the primary limitation of not just this study but most studies that use online public data. However, simultaneously, such data allows for anonymity and invisibility, which have been demonstrated to facilitate self-disclosure [74]. This is especially advantageous when studying emotions [31] and unmet needs.

### Conclusion

Cancer pain is an “emotional provoker” [4] that may drastically decrease the quality of life of patients and caregivers [7]. Therefore, it is crucial to consider the caregiver as part of pain management. As our study shows, they too are emotionally affected by the experience of their loved ones. Patients and caregivers are part of a common system, and taking care of the whole system could favor a better quality of life and pain relief for both. Within this study, we emphasize the importance of considering the perspectives of patients and caregivers. This allows identifying their needs and emotions that may affect pain management. Increasing knowledge among patients, caregivers, and health care providers is crucial for better pain management and decision-making processes. eHealth applications and technological infrastructure may help navigate the cancer journey; increase awareness of knowledge, needs, preferences, and expectations about treatments; and improve patient-doctor communication, empowerment, and involvement in the decision-making process [64,66].

Further studies are still needed to understand the interconnectedness of the behavioral and emotional reactions of caregivers and patients to cancer pain. Given that these reactions are formed in dyadic (or family) relationships (eg, patient-caregiver), dyadic analyses should be implemented to explore the mutual influence between two or more actors [75-77].

## Appendix

Multimedia Appendix 1 Distribution of posts based on the broad categories referencing patients’ and caregivers’ comments.

Multimedia Appendix 2 Word cloud comparison between patients’ and caregivers’ words used for describing the cancer pain experience. The words used by patients are in orange, and those used by caregivers are in green.

Multimedia Appendix 3 Patients’ emotion and sentiment frequencies. Sentiments and emotions associated with a negative affect are represented in red, and those associated with a positive affect are represented in green. Surprise may be associated with a positive or negative affect; thus, red and green are combined.

Multimedia Appendix 4 Caregivers’ emotion and sentiment frequencies. Sentiments and emotions associated with a negative affect are represented in red, and those associated with a positive affect are represented in green. Surprise may be associated with a positive or negative affect; thus, red and green are combined.

Multimedia Appendix 5 Dendrograms based on the posts of patients and caregivers, and comparisons between them.

## Abbreviations

STM: Systematic Transactional Model
