# Supplementary material for: Cancer Pain Experience Through the Lens of Patients and Caregivers: Mixed Methods Social Media Study
Source: JMIR Cancer. 2023 Jul 3;9:e41594. doi: 10.2196/41594 (PMC10365594; doi:10.2196/41594)
Supplement: Multimedia Appendix 1 [file cancer_v9i1e41594_app1.docx]

**Multimedia Appendix 1.** Distribution of posts based on the broad categories referencing patients’ and caregivers’ comments.

| **Broad categories** | **Code** | **Example of comments** | | **Frequency (%)** | |
| --- | --- | --- | --- | --- | --- |
|  |  | Patients | Caregivers | Patients (n=518) | Caregivers (n=161) |
| **Pain Dimension** | Physical | *“[…] I have stage IV colon cancer which metastasized into my bones. The pain from bone cancer is crazy bad. I would shake and vomit due to how bad the pain would get….” P52* | *“My Aunt has stage IV. She's been on Keytruda for about a year now. Her symptoms were loss of appetite, vomiting, and tremors. The symptoms suck but her last MRI showed shrinkage in her main tumor and completely gone in the smaller ones that were giving her back pain […].” C8* | 359(69) | 50(31) |
|  | Psychological | *“[…] As for me, I have a recurrence of liver cancer. This time it's inoperable…I told my immediate family because we are close knit family. I told my best friend, and 2 other friends I can trust. I made the decision not to tell anyone else because I don't want to be treated differently. Also, when you drop the cancer bomb on people, you then have to comfort THEM instead of them comforting you. I can't deal with other people's emotions right now. For the first time in my life, I am being selfish and doing things my way […].” P468* | *“My dad had prostatic cancer in 2016, he did surgery and radiotherapy. Last year the PSA levels started rising again. It took them 1 year (pandemic did not help) to realize that it might now be in the bones, so prognostic is not good. Apart from my dad who is pessimistic by nature (and so am I) out of everyone in the family I feel like I'm the one really suffering, I cry every night, I cannot sleep. My brothers seem relaxed. I don't know how I am going to handle seeing my dad in pain and getting worse and worse […].” C37* | 37(7) | 44(27) |
|  | Both^a^ | *“[…] talk about anxiety and make sure you don't apologize or downplay it. Be anxious. Show anxiety. That doctor can refer you to an assortment of care providers AND some of them may be telemed so that helps me. Just getting to a doctor exhaust me….Cancer itself causes pain. Acute, scale of 7-10, needs immediate attention. Cancer treatment causes pain. Like chemo and neulastin. Radiation therapy causes still more. Nerve damage. And some time along the path, it might be acute, or drop down to moderate, scale 4-7, chronic, maybe it won't heal so how to manage it […]. Ask for help. There are good sleep meds to help. And for talk therapy there are cancer specific psychologists. Make a list, then make them listen. If it's too much for you, bring your best advocate. Family. Best friend. Someone who knew you before your illness and can speak with truth about the old you and how cancer has changed you[...].” P104* | *“[…] My husband just passed away from his cancer which was diagnosed three years ago. It became stage iv two years ago…The prognosis is really just hopeless once it metastasizes. The day his routine checkup scan two summers ago revealed the inoperable metastasis in his lungs, has been the most devastating day of our lives. Far worse than the day he passed away. Because it changed our lives as we knew it. There was no going back to the “normal life”. Living with cancer is a whole change in lifestyle […].*  *Whenever we had breakdowns after a bad news or he wasn’t feeling well, I was there to listen to him and empathize with him as he expressed the different types of pain he was feeling. We both knew I couldn’t cure his symptoms, but I did what I could […] ” C376* | 123(23) | 67(42) |
| **Type of comments** | Advice | *“I also had weird pain in my neck too and strangely my arm when I first got it. My advice is to fight through that weird discomfort and turn your neck/move your arm. It’s normal for it to feel stiff because of the catheter placement, but when you keep moving your neck and arm it saves you trouble for doing extra physio for it in the future.” P359* | *“Your own knowledge of your body should trump what the docs say. You've known it for a lot longer, and a lot more in detail than they can ever hope to… I’m not sure where you're located, or your access to healthcare. If it were me, or my loved ones, I’d recommends: see a specialist (Ear/nose/throat ask to otolaryngologist) and get the 'lump' in your throat checked. By seeing a brand-new doctor, they are more likely to work up a complete test, as opposed to a GP/family physician that sees you fairly frequently […]” C786* | 53(10) | 13(8) |
|  | Experience | *“I had this horrible pain for over a year that eventually got to the point where I was on some strong pain meds and not able to sleep because it would constantly wake me up. I don’t really know what I thought it was, but I never got checked out because I didn’t have insurance. Finally realized something was serious when I ended up in the ER because I lost complete movement and feeling from my chest down.” P5* | *“My mom was diagnosed with the same leukemia in January. I had a similar experience with her, she was diagnosed at the oncologist's office, and we admitted her to the hospital a few hours later. She's had three rounds of chemo and is now having her bone marrow transplant at Thomas Jefferson (she is having a half match donor transplant) […]. Chemo may not seem taxing now but it will probably get worse and she may not be up for the procedure of freezing her eggs. I'm not sure what that procedure is, but I've seen my mom go through 3 rounds of chemo and it's very tiring. […]. My mom complained a little bit about joint pain, but I haven't heard any doctor say that will be a lasting problem. My mom was in really great shape before she was diagnosed, she weights lifted and hiked all the time. But she's also 53 so her experience will probably be different than your wife's.[…]. ” C163* | 422(81) | 130(81) |
|  | Both^b^ | *“I can honestly say I have been in pain every day for 18 years. Please get painkillers! They are absolutely lifesaving! If your doctor won't prescribe any then go find a pain clinic and bring all your medical records…Cancer is becoming more and more of a chronic disease for some people rather than a death sentence. If your cancer is being kept stable and you still have a semi-decent quality of life (you're still able to travel drink alcohol!) then it's just something, you'll have to live with…I'm on a targeted therapy drug that just got developed less than 10 years ago. Unless there are *significant* advances in healthcare in my lifetime, I can't be cured and it's likely that I'll just have to take the targeted therapy for the rest of my life. I'm currently 20 and I've been taking it for a couple years…I'm expected to have a half-decent lifespan if I continue dealing with the side effects and can afford it…I need to learn to live with this as a chronic disease and stop worrying about a cure. It's not killing me right now, it's just stable, so I'll be fine unless something goes super wrong. But accidents can happen all the time, not just with my cancer. Worrying all the time about mine getting aggressive would be as pointless as me worrying about getting run over. Chronic pain is a SIGNIFICANT deterrent in having a good quality of life.” P222* | *“[…] On to your questions. I'll answer them in the order asked. My father did the BCG treatment while the tumor was at stage 1. This is the default first defense urologist and oncologists usually consider for early-stage bladder cancer. The moment the first tumor was discovered, surgery was done to get it out of him. They pulled a 3cm. tumor out that was shaped like a cauliflower. This was the only surgery done prior to BCG to remove tumors. Experience with BCG was unpleasant. The virus is administered via a tube slipped into the urethra. It has a bad burning and itching sensation. My dad was told to hold it in for at least 1-2 hours if I remember right. It made the drive home from treatment hard, especially if there were bumps in the road. After the allotted time limit, my dad was allowed to urinate. What followed after the first day of treatment would be flu like symptoms. Aside from those, no hair loss. Just some nausea and the itchy/burning pain in the bladder. Urinating was a bitter/sweet sensation as the affects wore off over weekends. The symptoms could last 2-4 days […]”C801* | 34(7) | 18(11) |
|  | Question | *“Did you have fatigue and neuropathic pain during all of Taxol? I'm doing 4x and have done 3, 3rd was today. Still haven't felt any side effects.” P282* | *NA* | 9(2) | 0 |
| **Type of pain** | Acute | *“[…]I know how it feels. My symptoms started with severe acute pancreatitis which sent me in an ambulance to the ER. Was admitted for 10 days and they removed my gall bladder. Gall stones had blocked the bile duct at the opening to the pancreas. After that I had periodic (about once a month) attacks of pain that typically required a visit to the ER because of severe pain.” P103* | *“I know it doesn't sound healthy, but my mom (75, mouth cancer) spent a LOT of days on just Boost and Chocolate Milk…She's currently having mouth discomfort from the chemo but nothing like she had from the radiation” C355* | 51(10) | 4(2) |
|  | Chronic | *“[…]We can affect our lives by making choices. Sometimes our choices are severely limited due to luck, chance and circumstance[...]. I’ve had cancer since I was 10 years old. I’ve had chronic pain for 15 years. I started balding when I was 15-16. My teeth took a huge toll during chemo. For 10 years my right leg was 4-5 inches shorter than my left, with my feet being 3 shoe sizes different from each other. I walked with a very noticeable limp. Then the cancer returned, and my leg was amputated. P252* | *“[…] It is hard to understand and accept that the process to solve chronic, cancer pain is complex, long, and difficult. They need to find a balance between keeping him alive and addressing his pain, and it's not easy. Trust the doctors but keep advocating for your dad. It's been five weeks for my wife and we're still working on it. One step forward and two backwards so far. In my wife's case, it was fundamental to understand anxiety and trauma from pain were making everything worse, were increasing her physical pain, or at least the perception of it, so including psychiatric medication is helping. As for those witnessing his pain: I'm sorry, I'm so sorry. I know how much it hurts and how much this affects you. It is extremely painful and difficult to live with. I am hugging you so tight. My heart is with you. My only advice is to take turns. Everyone experiencing this needs some distance from it from time to time. As much as it hurts and scares me to be away from my wife, I had to ask my Mil to come and stay with her at the hospital for a few days (I'll stay the nights). If I don't spend some time away from pain I will lose my mind, I know […]” C261* | 80(15) | 18(11) |
|  | Acute neuropathy | *“[…] I got 4 rounds of AC, spaced two weeks apart. I started getting sick at my stomach about 8hrs after treatment one. Then two days later felt sick all day. That pattern repeated for each of those courses. Felt sore for several days after each of these. The soreness was probably caused by the neulasta, not the actual chemo. Hair fell out around 3 weeks after first treatment. Then I had 16 weeks of taxol. I didn't get sick to my stomach or ill feeling but the effects like weakness, neuropathy, and bleeding nails got worse the more of them I got.” P590* | *“[…] Experience with BCG was unpleasant. The virus is administered via a tube slipped into the urethra. It has a bad burning and itching sensation. My dad was told to hold it in for at least 1-2 hours if I remember right. It made the drive home from treatment hard, especially if there were bumps in the road. After the allotted time limit, my dad was allowed to urinate. What followed after the first day of treatment would be flu like symptoms. Aside from those, no hair loss. Just some nausea and the itchy/burning pain in the bladder. Urinating was a bitter/sweet sensation as the affects wore off over weekends. The symptoms could last 2-4 days. C801* | 18(3) | 1(0.6) |
|  | Chronic neuropathy | *“Acute pain in my prostate area starting at the end of May 2010, became chronic over the next few months…the worst pain has been a result of radiation and chemo induced neuropathy. I have a dependency on oxycontin now because I had to take it through the course of my treatment along with morphine and dilaudid. Lost feeling in both my feet at one point due to the effects of vincristine (aforementioned neuropathy)[…]. Pain and feeling like death sums it up nicely. Stage IV rhabdomyosarcoma btw.” P166* | *“My husband is 65. Same diagnosis in May 2019. Did the 6 weeks of chemo and radiation followed about a month later by the esophagectomy. He actually did great. In hospital 6 days and no complications. Back to work 6 weeks later. Got the J tube out about 2 months after surgery. He was thin to start with so keeping weight on has been a struggle. They had to convert his laparoscopic procedure to open thoracotomy because when they went to separate his esophagus and trachea they were pretty well stuck and they made a hole in the trachea they had to repair urgently so converted to open. Still recovered really well though. Has had some persistent numbness and soreness right on the site of the big incision on his flank area […]. He just started Opdivo and Folfox today.” C554* | 52(10) | 3(2) |
|  | Neuropathy | *“[…] I also have incurable bone cancer (Ewing Sarcoma). Bone pain is so f* rough man. And I have neuropathy too. I’m gonna tell you my pain meds that I take every day.*  *Gabapentin (nerve pain)*  *Amitriptyline (nerve pain)*  *Hydromorphone (opioid for overall body/bone pain)*  *Celebrex (aleve works too) (NSAID drug which helps my bone pain)*  *I’m telling you those so that when you talk to a palliative/pain doctor, you can advocate for him and ask questions about more specific drugs if you need to.” P48* | *“[…] We tried Tube feeding, both NJ and J tube. I had so much hope on this last one. It didn’t work for him. He gets a lot of bloating and inflammation, even connected 24 hrs at a slow drip, he couldn’t tolerate it. The situation got worse with the leaking and skin burns. Doctor offered to try again, but I do think my dad is going through ptsd about it. He is suffering a lot just with the thought of it. I will try to bring it up again, I do almost every day, but he doesn’t want to go through it.” C300* | 95(18) | 12(7) |
|  | Somatic | *“[…] After my surgeries, I found it helpful to also work with someone who does somatic experiencing and somatic touch because of the trauma related to surgery and just the overall cancer experience […] ” P267* | *NA* | 2(0.4) | 0 |
|  | Visceral | *“[…] Cancer pain is like no other. It’s this visceral, holistic, constant overall pain no matter where it’s located. The mucositis from radiation is particularly painful and opiates are just about the only thing that allows you to eat and drink […] ” P270* | *NA* | 1(0.2) | 0 |
|  | Unknown | *“I couldn't sit still. Not hyperactive or anything, I just kept adjusting and moving in my seat. My doctor said it was probably the normal side effect of bone pain, but I never thought the pain was that bad, until he prescribed me some pain killers and I full on relaxed and was able to sit still […] ” P1* | *“[…] I’m a 22F. I don’t have cancer but my mom does—a very rare type of brain cancer. It came suddenly and at first, all I could think about was how unfair it was. Now I’m able to recognize all the amazing things that come along with cancer (never thought I’d say that). We are so blessed to have life, and to have people that love us. We aren’t guaranteed anything in life. Honestly, cancer opens up people’s eyes to an immense amount of beauty, and pain. Stay strong.” C34* | 219(42) | 123(76) |
| **Type of cancer^c^** | Blood^d^ | *“Just got through with chemo for Stage II B Hodgkin’s Lymphoma[…]” P33* | *“My boyfriend got Hodgkin’s lymphoma at the age of 30, and it was traumatic […]” C366* | 73(14) | 16(10) |
|  | Breast | *“Stage 4 Breast Cancer (HER2+)[…]” P67* | *“Caregiver for Stage IV Breast Cancer…” C49* | 25(5) | 7(4) |
|  | Gynecological^e^ | *“I've had cervical, vaginal and uterine cancer […]” P580* | *“Husband of 33F Stage 3 Ovarian Cancer Fighter[…]” C225* | 13(2) | 2(1) |
|  | Pancreatic | *“[…] Got pancreatic cancer[…] ” P14* | *“He has stage IV Pancreatic cancer […]”C96* | 6(1) | 8(5) |
|  | Melanoma | *“Stage 4 Melanoma […]” P190* | *“My mom had a melanoma tumor in T4 and T5 […]”C57* | 11(2) | 3(2) |
|  | Sarcoma | *“[…] I also have incurable bone cancer (Ewing Sarcoma)…” P48* | *“For my son it was his arm aching. He was 12 so I assumed growing pains. After 2 weeks, I could then feel a knot. Then our nightmare (osteosarcoma) began” C403* | 22(4) | 3(2) |
|  | Lung | *“I have stage IV non-small cell lung cancer […]. ” P240* | *“[…] My husband just passed away from his cancer which was diagnosed three years ago. It became stage iv two years ago. It’s a very very rare type of cancer. The prognosis is really just hopeless once it metastasizes. The day his routine checkup scan two summers ago revealed the inoperable metastasis in his lungs[…].” C376* | 5(1) | 9(6) |
|  | Colorectal | *“They found a 4mm spot on my liver (from my colorectal cancer) and want to do ablation this week to destroy it […]. ” P820* | *“Caregiver to Stage IV colon cancer spouse[…].” C610* | 19(4) | 3(2) |
|  | Brain | *“Have brain cancer, its in my motor/sensation cortex […].”P753* | *“[…] I’m a 22F. I don’t have cancer but my mom does—a very rare type of brain cancer […]. ” C34* | 14(3) | 5(3) |
|  | Others | *“[…] I'm stage IV Appendix cancer or more specifically Pseudomyxoma Peritonei […] .” P653* | *“Thank you thank you my friend, Brachytherapy is an option to save his bladder […]” C74* | 53(10) | 16(10) |
|  | Not diagnosed | *“[…] I’m not searching for a diagnosis due to anxiety, I’m searching for the reason for my 7/10 pain every day. The radiologist already told me that the mass on my lung could be malignant and I am seeing him next week for a follow-up scan […] ” P186* | *NA* | 7(1) | 0 |
|  | NA | *“I couldn't sit still. Not hyperactive or anything, I just kept adjusting and moving in my seat. My doctor said it was probably the normal side effect of bone pain, but I never thought the pain was that bad, until he prescribed me some pain killers and I full on relaxed and was able to sit still. It was my mum who said she noticed how suddenly I was sitting still.” P1* | *“I am caring for my mother right now and what I would my friends to do is something I haven’t found a way to ask them. I think of my mom every day at every moment, but some of my friends don’t want to ask or inquire as much as they might be interested in, because they think it will upset me. So they ask about other things in my life and sometimes point out the good parts, but none, and I mean NONE, of it matters when your parent has cancer. I would want my friends to be comfortable with the situation. To be able to ask and sit with the silence when I say that I feel tremendous pain or weight on myself. I don’t need them to lift the pain away. But just be there with me, weather those are texts or calls or meet ups. I would want constant reminder that they are there. Because even when they say they are and that I can absolutely reach out, I need them to do the reaching. So from my situation no gifts or food deliveries wouldn’t help. It’s the presence of a true friendship I need.” C63* | 270(52) | 89(55) |

*Note.* ^a^ physical and psychological; ^b^advice and experience; ^c^type of cancer of patients discussed in the posts; ^d^leukemia, lymphoma, myeloma; ^e^ovarian, cervical, uterine, vaginal, vulvar; NA=not available.
