# Supplementary figures and images for: Cancer Pain Experience Through the Lens of Patients and Caregivers: Mixed Methods Social Media Study
Source: JMIR Cancer. 2023 Jul 3;9:e41594. doi: 10.2196/41594 (PMC10365594; doi:10.2196/41594)

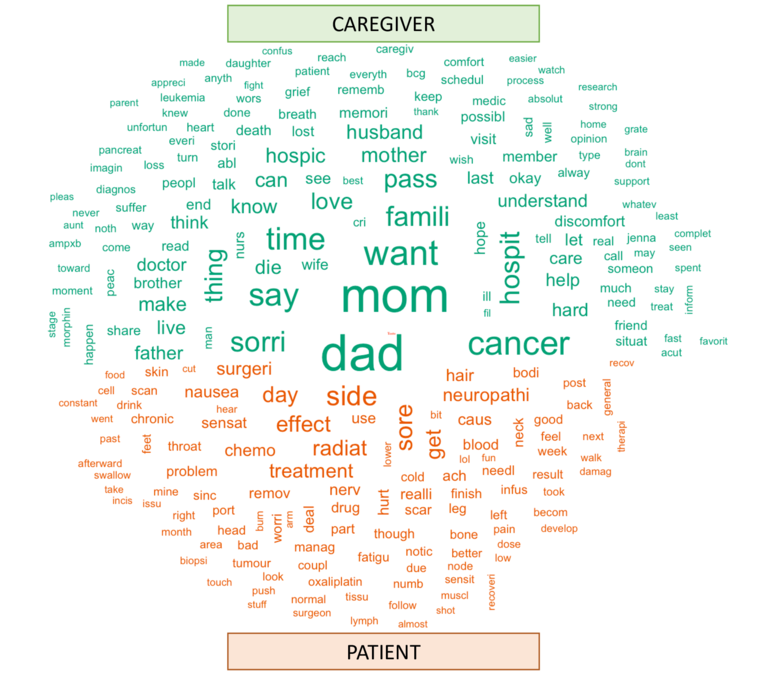

Supplement: Multimedia Appendix 2 [file cancer_v9i1e41594_app2.png]

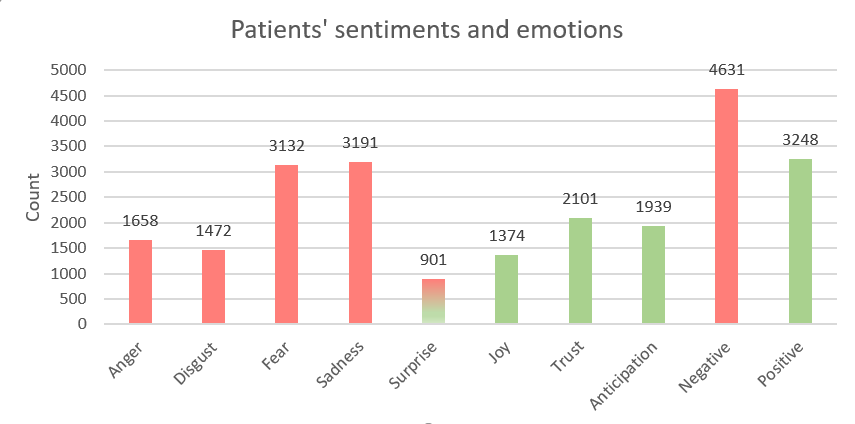

Supplement: Multimedia Appendix 3 [file cancer_v9i1e41594_app3.png]

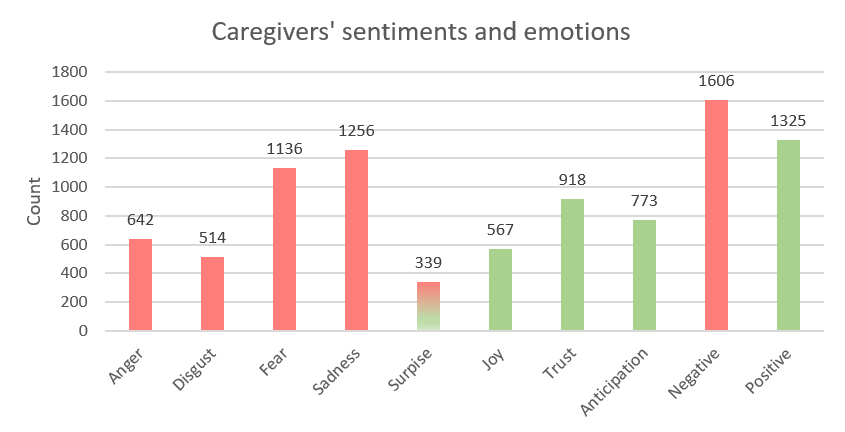

Supplement: Multimedia Appendix 4 [file cancer_v9i1e41594_app4.png]

## Slide 1
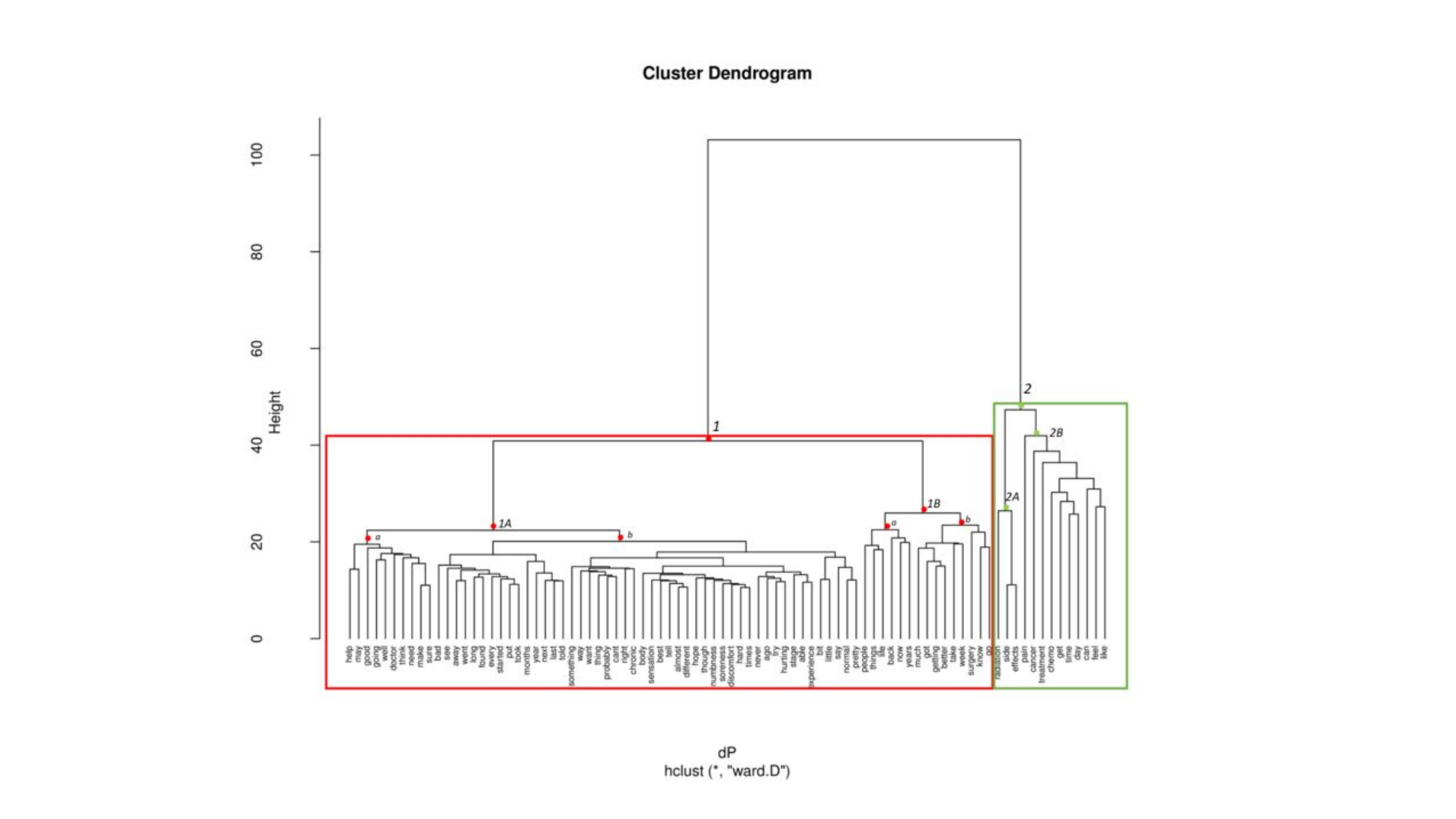

## Slide 2
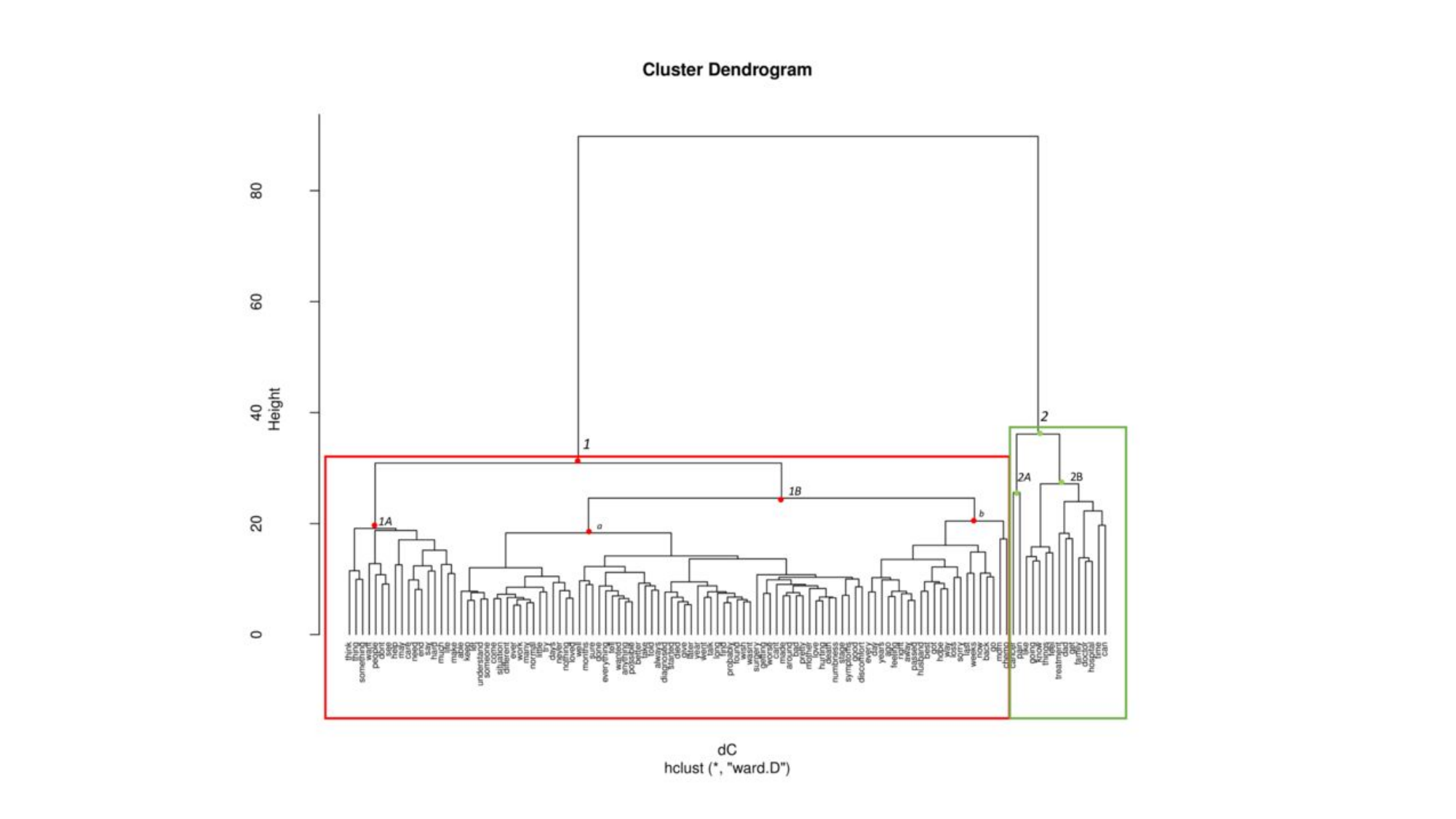

## Slide 3
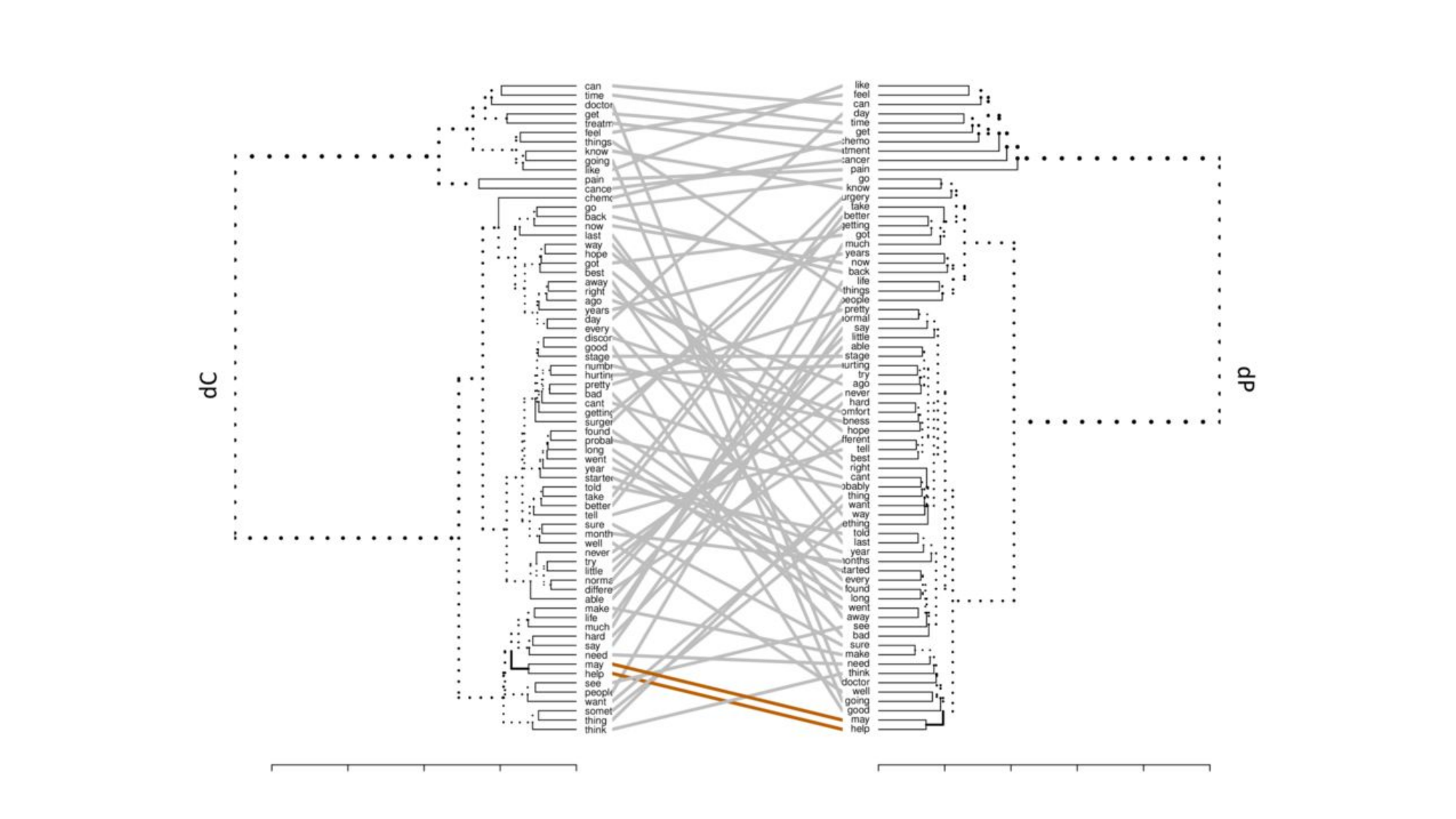

Supplement: Multimedia Appendix 5 [file cancer_v9i1e41594_app5.pptx]
